# Permanent Military Hospitals

**Published:** 1900-10-13

**Authors:** 


### EDITOR'S LETTER-BOX.

[Our Correspondents are reminded that prolixity is a great bar to publication, and that brevity of style and conciseness of statement greatly facilitate early insertion.]

### PERMANENT MILITARY HOSPITALS.

THE MEDICAL SUPERINTENDENT OF THE BROOK HOSPITAL writes: My attention has been directed to an article on Permanent Military Hospitals in your issue of 6th inst. The Brook Hospital is contiguous to the Military Hospital at Woolwich, and the writer of the article makes certain observations reflecting on the nursing staff of the Brook Hospital. The nurses here resent most strongly the statements of your contributor, which constitute an aspersion upon them. On their behalf I beg to protest in the strongest possible manner against the unwarranted and uncalled-for observations contained in the article. As regards the leave of absence given to the nursing staff here, your contributor appears to be under the impression that this is given on special days. That is not the case; leave is given daily to a proportion of the nurses. I shall be glad if you will insert this letter in your next issue.

[If our correspondent will read the article again he will perceive that the writer of it does not make any aspersions on the nurses of the hospital referred to, nor does he even mention them. We are confident the writer of the article did not intend to refer to these nurses. Much, moreover, may be undesirable from a disciplinary point of view which may be perfectly "correct" both ethically and socially.—Ed. Hospital.]

